# Translation to Brazilian Portuguese, cultural adaptation and psychometric properties of 8-item ArthritisSelf-Efficacy Scale (ASES-8)

**DOI:** 10.1590/1516-3180.2018.0354071218

**Published:** 2019-05-08

**Authors:** Raphael Vilela Timoteo da Silva, Fabiana de Carvalho Silva, Sandra Mara Meireles, Jamil Natour

**Affiliations:** I PT, MSc. Doctoral Student, Rheumatology Division, Universidade Federal de São Paulo (UNIFESP), São Paulo (SP), Brazil.; II PT, MSc. Doctoral Student, Rheumatology Division, Universidade Federal de São Paulo (UNIFESP), São Paulo (SP), Brazil.; III PT, PhD. Physiotherapist, Rheumatology Division, Universidade Federal de São Paulo (UNIFESP), São Paulo (SP), Brazil.; IV MD, PhD. Associate Professor, Rheumatology Division, Universidade Federal de São Paulo (UNIFESP), São Paulo (SP), Brazil.

**Keywords:** Translations, Validation studies, Self-efficacy, Arthritis, rheumatoid, Surveys and questionnaires

## Abstract

**BACKGROUND:**

Self-efficacy refers to one’s belief in one’s ability to organize, perform actions and face challenges in order to achieve goals and motivation. High self-efficacy improves disease coping and adherence to treatment among patients with rheumatoid arthritis. The objective of this study was to translate, culturally adapt and test the reproducibility of the 8-item Arthritis Self-Efficacy Scale (ASES-8) questionnaire for use in Brazil.

**DESIGN AND SETTING:**

Validation study conducted in university outpatient clinics.

**METHODS:**

The questionnaire was translated into Brazilian Portuguese and then back-translated into English. The final version in Portuguese was tested on 30 patients with rheumatoid arthritis and was shown to be understandable and culturally adapted. A further 32 patients with rheumatoid arthritis were evaluated three times using the questionnaire. On the first occasion, two evaluators applied the questionnaire to check inter-evaluator reproducibility. After 15 days, one of the evaluators reassessed the patients to verify intra-evaluator reproducibility. At the first assessment, to test the construct validity of ASES-8, the Numerical Pain Scale, Health Assessment Questionnaire, Beck Depression Inventory and Short Form-36 questionnaire were also applied to all the patients.

**RESULTS:**

The inter and intra-evaluator correlation coefficients for ASES-8 were high. Cronbach’s alpha was higher than 0.90 for the questionnaire, indicating excellent internal consistency. There were moderate correlations between ASES-8 and most of the instruments tested, indicating good construct validity.

**CONCLUSION:**

ASES-8 was translated and adapted to the Portuguese language for Brazil. This instrument is valid, reproducible and reliable for evaluating self-efficacy among patients with rheumatoid arthritis.

## INTRODUCTION

Self-efficacy refers to a person’s beliefs in their ability to organize, perform actions and face challenges in order to achieve aims and motivation.^1^^,^^2^ It is not a matter of possessing certain capacities but is a belief that one has them or that one can acquire them through personal efforts (outcome expectancy). The strength of individuals’ self-efficacy has an effect on how much effort and perseverance they will apply to achieve an aim.^3^

Rheumatoid arthritis is a chronic autoimmune inflammatory disease characterized by pain and destruction of synovial joints that may lead to disability.^4^ Epidemiological studies have estimated that the prevalence of rheumatoid arthritis in the adult population is 1%. It affects women three times as much as men and its incidence is highest among people aged between 35 and 65 years.^5^

Several studies have found that among patients with rheumatoid arthritis, greater self-efficacy is a predictor for healthy behaviors, such as physical activity, healthy eating and strategies for dealing with pain.^6^^,^^7^^,^^8^^,^^9^ Greater self-efficacy has also been correlated with lower daily pain, better emotional states, less stiffness, better functional capacity, better physical and mental wellbeing, less depression and better adherence to medication and other health recommendations.^10^ It has also been associated with better health outcomes, including physical activity recommendations for rheumatoid arthritis patients.^6^^,^^7^^,^^11^ In a recent review of the literature, negative correlations were found between self-efficacy and disability, pain, fatigue and duration of disease.^12^ Studies have also suggested that self-efficacy is associated with the health outcomes of people with rheumatoid arthritis. In these studies, it was observed that the higher the self-efficacy was (which can be changed through educational programs), the higher the association that the patients had with better health status.^13^^,^^14^^,^^15^

The 8-item Arthritis Self-Efficacy Scale (ASES-8) was created as part of a development process on a set of health assessment tools for the Spanish language. ASES-8 is derived from the full version of the Arthritis Self-Efficacy Scale (ASES), which has a total of 20items divided into three subsets. ASES-8 features two items from the ASES pain subscale, four items from the ASES other symptoms subscale and two new items relating to prevention of pain and fatigue that interfere with daily activities. Thus, it contains a total of eight items without a subscale. The responses to each of the items range from 1 (very uncertain) to 10 (very certain), such that higher scores indicate higher confidence or self-efficacy. The final score is the mean of the scores from the eight items.^10^

The original scale, in Spanish, was published in 1995.^16^ TheEnglish version of ASES-8 refers to both rheumatoid arthritis and fibromyalgia in each item.^17^ The German version was translated from the English version and was tested on both rheumatoid arthritis and fibromyalgia patients; in this German version, the term rheumatoid arthritis was replaced by fibromyalgia.^18^ ASES-8 has been shown to have good reliability, validity and adaptability, even when translated into several languages, such as English, German and Chinese.^10^^,^^17^^,^^19^

## OBJECTIVE

To translate, culturally adapt and test the reproducibility and construct validity of the ASES-8 questionnaire for use in Brazil, among patients with rheumatoid arthritis.

## METHODS

This validation study was conducted in two stages among a total of 62 patients. Firstly, the translated version of the questionnaire (used for translation and cultural adaptation) was administered to 30 patients to test their understanding of the tool. In the second stage, 32 patients were included to test the reproducibility and construct validity.

The sample size was determined as at least 30 patients for each phase, in accordance with the guidelines of Beaton etal.^20^ and Guillemin etal.,^21^^,^^22^ which have been used in other published studies to test the cultural validation and reproducibility of other questionnaires.

### Ethical considerations

This validation study was approved by our institution’s Ethics Committee (no. 907.062; date: December 9, 2014), and all participants gave their written approval before the evaluations weredone.

### Translation and cultural adaptation

Two English speakers who were Brazilian natives translated ASES-8 from English to Brazilian Portuguese as indicated by Beaton etal.^20^ and Guillemin etal.^21^^,^^22^ These two translators were English teachers. A committee composed of a rheumatologist and two physiotherapists reviewed the translation in order to reach an agreement on the Brazilian Portuguese version. Thisapproved version was then translated back into English by two other English teachers who were natives of English-speaking countries and had no knowledge of the original questionnaire. This back-translated version was then compared with the original questionnaire to ensure that it was semantically equivalent.

This Brazilian Portuguese version of ASES-8 (which was considered to be the test version) was then applied to 30 patients aged between 18 and 60 years who were selected from the outpatient clinic. All of these patients had presented rheumatoid arthritis (classified in accordance with the criteria of the American College of Rheumatology) for at least one year^23^ and had been undergoing treatment while in a stable condition for at least three months.

Patients with associated rheumatic, neurological or musculoskeletal diseases, patients who were unable to understand the Portuguese language and patients whose medication dose or treatment had been changed were excluded from this study. To assess cultural equivalence, the understanding level of the patients was measured through a yes/no answer to the question, “Do you understand what is being asked?” All items that were not understood by at least 20% of the respondents would be reviewed by the specialist committee and the revised version of the questionnaire would be retested on 30 patients.

### Reproducibility

After the Brazilian Portuguese version of ASES-8 had been tested and its semantic and cultural equivalence had been verified, a new group composed of 32 patients was selected with the same inclusion and exclusion criteria. These patients were evaluated three times, on two occasions. On the first occasion, two evaluators applied the questionnaire in separate interviews on the same day to verify inter-observer reproducibility. On the second occasion, between 7 and 15 days later, one of the evaluators reapplied ASES-8 in a single interview with the intention of verifying intra-observer reproducibility. The internal consistency was also evaluated.

### Construct validity

The construct validity was tested during the first interviews through simultaneous application of the following questionnaires: Numerical Pain Scale (NPS),^24^ Health Assessment Questionnaire (HAQ),^25^ Beck Depression Inventory (BDI)^26^ and Short-Form Health Survey (SF-36).^27^ The NPS evaluates pain through a numerical scale, on which patients quantify their degree of pain on a line from 0 to 10 centimeters, such that 0 represents absence of pain and 10 represents an unbearable pain.^24^ The HAQ evaluates the functional capacity of patients with rheumatoid arthritis using a total of 20 questions, from which the score is obtained by adding together the highest grades of each subscale. The scores range from 0 to 3, and the higher the resultant value is, the lower the functional capacity of the patient is.^25^ The BDI evaluates the depressive state of the patient through 21questions regarding how the individual felt in the last week. Each question has at least four possible answers (0to3). Theresult is obtained by summing the values of each question and is categorized thus: 0 to 13, no depression; 14 to 19, mild depression; 20 to 28, moderate depression; and 29 to 63, severe depression.^26^ The Short Form-36 (SF-36) evaluates the patient’s quality of life. It is divided into eight domains with 36 questions in total. The scores range from 0 (worst) to 100 (best), and the higher the score is, the better the patient’s quality of life is.^27^ Allof these instruments had previously been validated for use in Brazilian Portuguese. These instruments were chosen because the ASES-8 items correlate with the patient’s emotional state, pain and functional ability. To assess the versions of ASES-8 that were previously validated for use in German and Chinese, similar methods were used.^18^^,^^19^

### Statistical analysis

Descriptive analyses were used to demonstrate the data averages and standard deviations. Interclass correlation coefficients (ICCs) and Bland-Altman analyses were used to assess the inter and intra-observer reproducibility. Internal consistency was assessed using the Cronbach’s alpha test. Spearman’s correlation test was used to investigate the construct validity. Analyses were performed with assistance from the Statistical Package for the Social Sciences (SPSS) software, version 17.0 (Chicago,IL,USA).

## RESULTS

A total of 94 patients were invited to take part to the study, but 32 of them were not included because of the presence of exclusion criteria, thus leaving a total of 62 patients. Thirty patients were included in the cultural adaptation phase and 32 in the reproducibility and construct validity phase. There was no patient loss during the application of the study.

In the cultural adaptation phase, every question of the questionnaire was understood by more than 80% of the participants, and no item required review by the committee experts (Appendix1). Table 1 shows the demographic and clinical data of the patients who participated in the reproducibility and construct validity phase.

**Table 1.** Clinical and demographic characteristics of the patients in the reproducibility and construct validity phase

Variables	Total (n = 32)
Gender (n, %)	
Female	26 (81.3%)
Male	6 (18.7%)
Age (years)	53.8 (± 8.2)
Education (years)	7.2 (± 4.3)
Length of time with illness (years)	13.1 (± 6.9)
Employment situation (%)	
Employed	13 (40.3%)
Unemployed	7 (21.9%)
Retired	12 (37.5%)
Steinbrocker functional classification^28^ (%)	
1	10 (31.3%)
2	21 (65.6%)
3	1 (3.1%)
4	0 (0%)

Data are presented as average ± standard deviation or as percentage (%); n=number of patients.

Table 2 indicates that there were strong correlations between the results obtained in the intra and inter-observer assessments, with an ICC of 0.954 in the intra-observer assessment and an ICC of 0.972 in the inter-observer assessment (95% confidence interval).None of the patients changed their medication in the interval between the testing and retesting of the questionnaire. Table2also shows that the Cronbach’s alpha was higher than 0.90, thus indicating that the questionnaire had good internal consistency.

**Table 2.** Inter and intra-evaluator reproducibility and internal consistency of the 8-item Arthritis Self-Efficacy Scale (ASES-8)

	A1 Mean ± SD	A2 Mean ± SD	R1 Mean ± SD	ICC A1 vs. A2	ICC A1 vs. R1	Cronbach’s alpha
ASES-8	5.93 ± 2.19	5.73 ± 2.23	5.94 ± 2.04	0.954	0.972	0.985

A1 = evaluator 1; A2 = evaluator 2; R1 = re-evaluation of evaluator 1; ICC=interclass correlation coefficient; A1 versus A2 = inter-rater evaluation; A1 versus R1 = intra-rater evaluation; SD = standard deviation; vs. = versus.

**Figure 1.**
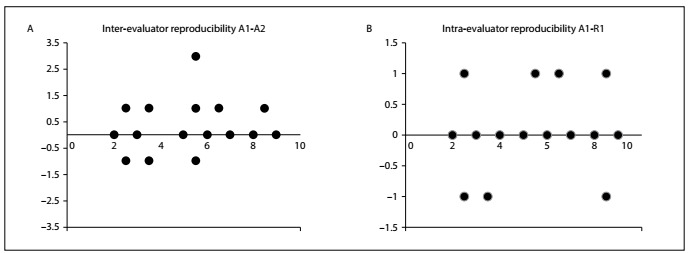
Bland-Altman reproducibility graphs: (A) inter-evaluator reproducibility; (B) intra-evaluator reproducibility.

Figure 1 illustrates the strong intra and inter-observer correlations of the questionnaire through Bland-Altman plots, which show that the average difference was always close to zero. Table3 indicates the correlation between ASES-8 and the other instruments, i.e. the HAQ, BDI, SF-36 and NPS. Moderate correlations were found between ASES-8 and the other instruments except for NPS, which did not show any statistically significant correlation with ASES-8.

**Table 3.** Spearman correlations between ASES-8 and NPS, HAQ, BDI and SF-36, to assess construct validity

ASES-8	NPS	HAQ	BDI	SF36 PF	SF36 PRF	SF36 BP	SF36 GHP	SF36 VIT	SF36 SRF	SF36 ERF	SF36 MH
R	- 0.278	- 0.437	- 0.562	0.545	0.321	0.427	0.473	0.445	0.435	0.376	0.558
P	0.12	0.01	0.001	0.001	0.04	0.01	0.006	0.01	0.01	0.03	0.001

Data are presented as r = correlation coefficient and P = significance; ASES-8=8-item Arthritis Self-Efficacy scale; HAQ = Health Assessment Questionnaire; BDI= Beck Depression Inventory; NPS = numerical pain scale; SF-36 = Short Form-36; PF = physical functioning; PRF = physical role functioning; BP = bodily pain; GHP = general health perception, VIT = vitality; SRF = social role functioning; ERF = emotional role functioning; MH = mental health.

## DISCUSSION

Self-efficacy, which can be altered through educational programs, is related to a specific behavioral characteristic and is strongly associated with health improvement and reduction in healthcare costs, thereby playing a key role in patients’ adaptation to chronic disease.^10^^,^^29^ Self-efficacy can potentially explain the discrepancy between possessing a skill and the ability to perform a task using that skill. Belief in self-efficacy predicts motivation levels, opinion standards, moods, emotional reactions and attitudes. Therefore, it is important to measure self-efficacy among patients with chronic disease.^30^

An increasing number of questionnaires are used to evaluate self-efficacy among patients with chronic conditions, and these questionnaires include ASES-8. A recent review of the literature indicated that ASES-8 provides high reproducibility, validity and responsiveness, thus making it highly recommended for evaluation of self-efficacy among patients with rheumatoid arthritis.^10^

There are two possible ways to make a questionnaire viable in a specific language: create a questionnaire for a particular ethnic group or translate and validate a questionnaire previously developed for use in another language. This second option, besides being less expensive in terms of time and resources, enables comparison of data collected in different countries.

ASES-8 includes a total of 8 items without any subscale, and higher scores equate to higher confidence or self-efficacy.^16^ Theadvantage of the ASES-8 questionnaire is that it specifically evaluates self-efficacy among rheumatoid arthritis patients, through focusing on the essential issues and characteristics of these patients, unlike the broader focus of general self-efficacy questionnaires, such as the General Self-Efficacy Scale (GSES).^31^

We followed the validation process described by Beaton etal*.*^20^and Guillemin etal*.*^21^^,^^22^ and obtained results with high internal consistency (Cronbach’s alpha of 0.985). Because rheumatoid arthritis is a chronic disease, we chose a two-week test-retest interval because we believed that over this period, there would be no significant changes in the disease state, and sufficient time would have elapsed for the patient to have forgotten the content of the first interview. None of the patients changed their medication during the test-retest interval. The intra and inter-observer ICC values were high (0.972 and 0.954, respectively).

To assess the construct validity, we compared the Portuguese version of ASES-8 with the NPS, HAQ, BDI and SF-36. Moderate correlations were found between ASES-8 and the HAQ and BDI tests and between ASES-8 and the majority of the SF-36 domains. However, comparison between ASES-8 and the NPS did not show any significant correlation. One reason for this might be that NPS assesses pain in an overall manner and at the current time, rather than in a way that specifically addresses the daily routine activities of rheumatoid arthritis patients, as is done in the three questions involving pain in the ASES-8 questionnaire. According to Barlow etal. and Mueller etal., these findings also can be explained by the fact that some people with rheumatoid arthritis feel that they have highly effective coping mechanisms, in relation to pain, regardless of the intensity of the pain. Conversely, some individuals with relatively low levels of pain may feel that they have little control over this symptom. Another explanation is the fact that patients with rheumatoid arthritis can increase their pain control through medication, thereby reversing the influence of pain on self-efficacy.^17^^,^^18^ Another factor that may explain the lack of correlation is that the behavior of the pain correlates not only with the intensity of the pain but also, in a major way, with the patient’s emotional state.

One limitation of our study was the low number of male patients, which can be explained by the higher prevalence of rheumatoid arthritis among women. However, this limitation will not prevent use of ASES-8 among men, given that it was developed to be applied to both genders. Another limitation was that we did not analyze criterion validity during the study.

## CONCLUSION

The 8-item Arthritis Self-Efficacy Scale (ASES-8) questionnaire was translated and adapted for use in Brazilian Portuguese. Thisquestionnaire is a valid, reproducible and reliable instrument for evaluating self-efficacy among patients with rheumatoid arthritis.
